# Disparities in dialysis modality decision-making using a social-ecological lens: a qualitative approach

**DOI:** 10.1186/s12882-022-02905-5

**Published:** 2022-08-05

**Authors:** Miriam Vélez-Bermúdez, Jenna L. Adamowicz, Natoshia M. Askelson, Susan K. Lutgendorf, Mony Fraer, Alan J. Christensen

**Affiliations:** 1 Department of Psychological & Brain Sciences, University of Iowa, Iowa City, IA USA; 2 Department of Community and Behavioral Health, University of Iowa, Iowa City, IA USA; 3 Department of Obstetrics & Gynecology, University of Iowa, Iowa City, IA USA; 4 Department of Urology, University of Iowa, Iowa City, IA USA; 5 Holden Comprehensive Cancer Center, University of Iowa, Iowa City, IA USA; 6 Department of Internal Medicine, University of Iowa, Iowa City, IA USA; 7 Department of Psychology, East Carolina University, Greenville, NC USA

**Keywords:** Decision-making, Qualitative methods, Dialysis, Healthcare disparities

## Abstract

**Background:**

Patients with end-stage kidney disease (ESKD) may choose to undergo dialysis in-center or at home, but uptake of home dialysis in the US has been minimal despite its benefits over in-center dialysis. Factors that may have led patients to select home dialysis over in-center dialysis are poorly understood in the literature, and interventions to improve selection of home dialysis have focused on patient knowledge and shared decision-making processes between patients and providers. The purpose of this study was to explore micro- and macro-level factors surrounding dialysis modality decision-making among patients undergoing in-center and home dialysis, and explore what leads patients to select home dialysis over in-center dialysis.

**Methods:**

Semi-structured qualitative interviews were conducted in a dialysis clinic at a large Midwestern research hospital, from September 2019 to December 2020. Participants were 18 years or older, undergoing dialysis for ESKD, and had the cognitive ability to provide consent. Surveys assessing demographic and clinical information were administered to participants following their interviews.

**Results:**

Forty patients completed interviews and surveys (20 [50%] in-center dialysis, 17 [43%] female, mean [SD] age, 59 [15.99] years). Qualitative findings suggested that healthcare access and engagement before entering nephrology care, after entering nephrology care, and following dialysis initiation influenced patients’ awareness regarding their kidney disease status, progression toward ESKD, and dialysis options. Potential modifiers of these outcomes include race, ethnicity, and language barriers. Most participants adopted a passive-approach during decision-making. Finally, fatigue, concerns regarding one’s dialyzing schedule, and problems with fistula/catheter access sites contributed to overall satisfaction with one’s dialysis modality.

**Conclusions:**

Findings point to broader factors affecting dialysis selection, including healthcare access and racial/ethnic inequities. Providing dialysis information before entering nephrology and after dialysis initiation may improve patient agency in decision-making. Additional resources should be prioritized for patients of underrepresented backgrounds. Dialysis decision-making may be appropriately modeled under the social-ecological framework to inform future interventions.

**Supplementary Information:**

The online version contains supplementary material available at 10.1186/s12882-022-02905-5.

## Introduction

To sustain life, patients with end-stage kidney disease (ESKD) require renal replacement therapy to take the place of their damaged kidneys. Dialysis, the most common form of renal replacement therapy, comprises 97% of incident ESKD cases and 70% of prevalent ESKD cases in the United States (US). Dialysis may be conducted in-center (i.e., in-center hemodialysis) or at home (i.e., peritoneal dialysis (PD) and home hemodialysis) [1]. In-center hemodialysis is administered by a nurse or a dialysis technician within a clinic setting, three times a week, 3–5 h at a time [1, 2]. In the US, PD is self-managed and conducted at home, and home hemodialysis requires a care partner to administer dialysis to the patient [1, 3, 4].

US patients undergoing home dialysis experience benefits such as flexible dialysis schedules, reduced need for transportation to clinics, and greater self-reported quality of life [1, 3–5]. In addition, Medicare cost savings per-patient-per-year is over $14,000 for PD compared to hemodialysis [1]. Although medical eligibility for in-center hemodialysis and home hemodialysis is comparable, patients who undergo home hemodialysis still experience quality of life benefits [1, 4, 5]. However, home hemodialysis is the least utilized form of dialysis, with about 1.3% of prevalent ESKD cases undergoing home hemodialysis [1].

Ideally, the decision for which dialysis modality to undergo should mostly depend on patient preferences, and occur within a shared decision-making context with a nephrology provider, in which nephrology providers inform and advise patients on their treatment options to facilitate a collaborative approach with patients to reach a final dialysis decision that aligns with patient needs and preferences [6–8]. However, despite benefits associated with home dialysis, prevalence rates for patients on dialysis in the US are overwhelmingly skewed towards in-center (87.5%) over both home dialysis modalities combined (12.5%) [1]. Given these rates, there may be eligible patients who would potentially prefer home dialysis, but are instead served in center.

While many factors may influence dialysis modality decision-making, past interventions and clinical recommendations aimed at supporting patients who must choose a renal replacement therapy that is right for them have focused on educating patients about their options and promoting shared decision-making between patients and nephrology providers [9, 10]. However, interventions intended to increase patient knowledge about treatment options, and to improve the shared decision-making process between healthcare providers and patients almost always occur right before the end stage of the disease is reached [11–16]. In other words, conversations between healthcare providers and patients regarding their options often occur too late to be effective or do not occur at all. In addition, efforts to improve patient knowledge about treatment options and shared decision-making processes have not generated enough change, and have not involved patients with early-stage chronic kidney disease (CKD) [13, 15–18]. Taken together, these factors may suggest that deficiencies in shared decision-making processes, and the overall low rates of home dialysis, are beyond the control of just patients and nephrology providers. Using a social-ecological lens to examine this phenomenon may help clarify what circumstances lead patients to be informed about their dialysis options.

The Social Ecological Framework (SEF) is used to understand the dynamic between various personal and environmental factors [19]. Under the SEF, the individual-, interpersonal-, organizational/community-, and policy/national-levels are all considered. In health-related research, this framework recognizes that while individuals have agency in making their own health-related decisions, their environment can either help or hinder the decision-making process as well as its outcomes [20, 21]. For example, relying on an individual patient approaching ESKD to either independently educate themselves about their dialysis options or find opportunities for dialysis education on their own dismisses the important role that nephrology providers could play in guiding them to a decision that works best with their overall health. Similarly, expecting shared decision-making processes to be the sole method in which optimal dialysis outcomes are reached ignores outside factors that may prevent patients from being referred to nephrology with enough time before they reach ESKD.

Little is known regarding the extent to which organizational/community- and/or policy/national-level factors, otherwise known as macro-level factors (e.g., lack of CKD screening recommendations, lack of hospital access to decision aid materials) affect patients’ dialysis modality decisions. Exploring dialysis modality decision-making through a social-ecological lens may shed light on challenges and barriers during the decision-making process that influence lack of patient knowledge and lack of a shared decision-making process.

The purpose of this qualitative study was to explore both micro- and macro-level factors surrounding dialysis decision-making among patients undergoing in-center and home dialysis to examine what factors influence the final dialysis decision as well as what leads patients to select home dialysis over in-center hemodialysis, henceforth referred to as “in-center dialysis.”

## Methods

### Design, participants, and setting

The present study employed an exploratory qualitative approach using in-depth individual interviews [22, 23] that later underwent content analysis [24]. This study design was utilized because factors associated with the dialysis decision-making process, such as macro-level factors, are largely unexplored. The Consolidated criteria for Reporting Qualitative Research (COREQ) checklist was used and reported in the supplementary material [25].

Eligible participants were 18 years or older, undergoing dialysis for ESKD, and had the cognitive ability to provide consent. A purposive sampling approach was employed, using referrals from nephrology providers. A diverse sample with respect to racial/ethnic background, rural/urban status, and time since dialysis initiation was recruited while achieving equal numbers of patients undergoing in-center and home dialysis.

Patients already undergoing dialysis were selected as the ideal candidates to shed light on dialysis modality decision-making processes, in lieu of patients who were approaching ESKD, because the former group had already undergone the decision-making experience, and the aim of the present study was to explore factors related to a final dialysis decision without interfering. Furthermore, both typical case sampling and intensity sampling were utilized [26, 27]. Typical case sampling is conducted to illustrate the average experience of participants (i.e., patients with ESKD undergoing in-center dialysis) [27]. Intensity sampling is a form of positive deviant case sampling, which is used to highlight cases that are uncommon but not necessarily unusual [26]. The purpose of this sampling strategy was to learn from “successful” cases, because these patients may have knowledge and insights regarding resources or strategies that could be applied to others. In this study, patients who were undergoing home dialysis were considered intensity cases, since home dialysis is vastly underutilized in the US [1].

Qualitative interviews were conducted with patients in a nephrology clinic at an academic medical center in the Midwestern region of the US. In-center dialysis patients participated during their dialysis session, and home dialysis patients participated in a private clinic room before or after their monthly appointments with nephrology providers. Home dialysis patients were only seen in the clinic once a week. Both patients undergoing PD and home hemodialysis were recruited from the home dialysis group. Every participant received $25 in appreciation of their time. All data were deidentified and the study was approved by the university’s institutional review board.

### Data collection

Semi-structured, individual interviews were conducted from September 2019 to December 2020 in English and Spanish (MVB) followed by a short questionnaire assessing demographic and clinical factors. There was a three-month hiatus in recruitment from March 2020 to June 2020 due to the COVID-19 pandemic. Most patients approached for recruitment agreed to participate. All patients undergoing home dialysis who were approached agreed to participate. About 75% of approached patients undergoing in-center dialysis agreed to participate. The primary reported reason patients declined participation was because they felt too weak to participate while they were dialyzing. Before participation, the interviewer introduced herself, described the study aims, and explained what participant involvement entailed.

The interview guide was informed by literature review of dialysis decision-making and common healthcare-related determinants outlined by the SEF [21]. The guide contained questions covering topics in the following areas: 1) participants’ journeys while learning they had CKD; 2) their pre-nephrology healthcare providers; 3) their accessibility to healthcare services prior to nephrology care; 4) their transition into nephrology care; 5) learning about their dialysis options; 6) how they made a final decision; 7) perceptions of their current dialysis modality; and 8) what they would have done differently during this process. Given that most interviews were conducted during the COVID-19 pandemic, participants were also asked how the pandemic had affected their lives as patients on dialysis. Although the primary topics remained the same throughout the study, questions and prompts of the interview guide underwent an iterative process based on what came up in interviews and how participants responded to questions. When necessary, participants were probed to clarify and provide more detail. Interviews were audio recorded and transcribed verbatim.

Questionnaire items asked participants to report their age, gender, race/ethnicity, relationship status, years of education attained, household income, zip code, general health status rating, comorbid diseases, and time since their CKD diagnosis, ESKD diagnosis, and dialysis initiation. Zip code was used to measure rurality using US Department of Agriculture Rural Urban Commuting Area codes [28, 29].

Sample size was guided by data saturation, meaning recruitment stopped once interviewing more participants did not provide additional insights regarding the study’s main objectives. In other words, recruitment ended once participants’ stories, which included different details, all began to allude to the same themes [30, 31].

### Analysis – Qualitative data

Interview transcripts were audited for clarity and removal of identifying information, then imported into Dedoose, a qualitative data analysis software package [32]. Qualitative analysis team members included two PhD candidates (MVB, JLA) and one faculty member (NMA). They had backgrounds in behavioral medicine, health psychology (MVB, JLA), clinical psychology (JLA), and community and behavioral health (MVB, NMA). All had advanced degrees, and two were experienced in qualitative research (MVB, NMA). Two analysis team members (MVB, JLA) used a blend of deductive then inductive-dominant content analysis [24]. The preliminary code list included some a priori codes based on the study aims, then other codes were added after reading the first few interview transcripts repeatedly and forming first impressions. Each interview transcript was analyzed independently using the code list (MVB, JLA), then jointly to resolve differences on codes and themes identified. The code list was iteratively adapted as interviews and analysis progressed. The third qualitative analysis team member (NMA) served as an advisor throughout the analysis process and provided input on codes and overarching themes. Qualitative analysis team members maintained rigor by record keeping within the code list, including code definitions, inclusion/exclusion criteria, adaptations, dates of changes, and examples using excerpts from participant responses. Overarching themes were derived from the finalized coded transcripts. Since data collection occurred in a single visit, transcripts and coded data were not returned to participants for correction.

### Analysis – Questionnaire data

Means, standard deviations, and ranges were calculated for continuous variables, and frequencies and percentages were calculated for categorical variables. All event information was recorded in years. If an event had occurred less than a year ago, it was recorded as a fraction out of 12 to convert to a year-value to obtain relevant descriptive statistics. Chi-square analyses and Pearson correlations were conducted to test within-sample differences between modality groups and significant findings are reported herein.

## Results

### Participant characteristics

Data saturation was reached at about 15 participants in each group, but five more participants per group were recruited. A total of forty participants (20 [50%] in-center hemodialysis, 17 [43%] female, mean [SD] age, 59 [15.99] years, 21 [53%] White, 30 [75%] with at least a high school diploma) completed interviews and short questionnaires. Eighteen participants in the home dialysis group were undergoing PD, and the other two were undergoing home hemodialysis. Fifteen participants lived in rural areas (38%), and only 6 (15%) participants were employed at least part-time during interviews. All Spanish-speaking patients of limited English proficiency within the clinic agreed to participate (*n* = 5). Interviews among the total sample lasted an average of 27.18 min (*SD* = 11.82) (Table 1).

**Table 1:** Demographic and clinical characteristics of total study sample (*N* = 40)

Characteristic (Reference group)	n (%)	Mean (SD)	Range
Age	-	59 (15.99)	29 – 89
Gender (Female)	17 (43)	-	-
Race & ethnicity (White)	21 (53)	-	-
Years of education	-	12.72 (3.90)	0 – 22
Relationship status			
Single *Includes: Single; Previously married, now widowed*	20 (50)		
In a relationship *Includes: Married; In a relationship, living with romantic partner; In a relationship, not living with romantic partner*	20 (50)		
Interview length [in minutes]	-	27.18 (11.82)	14.02 – 68.24
Currently employed (No)	34 (85)		
Rural status (Yes)	15 (38)		
Self-reported general health rating	-	2.87 (0.92)	1 – 5
Time since CKD diagnosis [in years]	-	10.07 (9.32)	0.42 – 36
Time since kidney failure diagnosis [in years]	-	4.55 (5.71)	0.08 – 27
Time since dialysis initiation [in years]	-	2.76 (2.46)	0.04 – 12
Diabetes	19 (48)	-	-
Hypertension	34 (85)	-	-

*Note*: 0.83 years = 10 months; 0.42 years = 5 months; 0.08 years = 1 month; 0.04 years = 2 weeks

### By modality

All Spanish-speaking participants were undergoing in-center hemodialysis. Fourteen of the Black and Latine participants were undergoing in-center dialysis (35%) [χ2(1, *N* = 40) = 8.42, *p* = 0.004], and thirteen of the rural participants were undergoing home dialysis (33%) [χ2(1, *N* = 40) = 14.02, *p* < 0.001]. On average, participants undergoing home dialysis had more years of education (*r*(38) = 0.41, *p* = 0.01) and were more likely to be in relationships (χ2(1, *N* = 40) = 6.58, *p* = 0.01) compared to patients undergoing in-center dialysis. Finally, patients undergoing home dialysis had comparatively better self-ratings of general health (*r*(39) = 0.37, *p* = 0.02) and initiated dialysis more recently (*r*(38) = -0.35, *p* = 0.03) (Table 2).

**Table 2:** Clinical and demographic characteristics by modality

*Characteristic (Reference group)*	**Home dialysis (*n* = 20)**			**In-center dialysis (*n* = 20)**			
	*n (%)*	*Mean (SD)*	*Range*	*n (%)*	*Mean (SD)*	*Range*	*p-value*
Age	-	59.40 (14.89)	31 – 85	-	58.60 (17.39)	29 – 89	0.88
Gender (Female)	8 (20)	-	-	9 (23)	-	-	0.75
Race & ethnicity (White)	15 (38)	-	-	6 (15)	-	-	0.004
Years of education	-	14.21 (2.70)	9 – 22	-	11.06 (4.42)	0 – 16	0.01
Number of family members per household		2.05 (0.85)	1 – 4		2.50 (1.36)	1 – 6	0.23
Marital status (Single)							0.01
Single *Includes: Single; Previously married, now widowed*	6 (15)			14 (35)			
In a relationship *Includes: Married; In a relationship, living with romantic partner; In a relationship, not living with romantic partner*	14 (35)			6 (15)			
Interview length [in minutes]	-	28.26 (13.69)	14.42 – 68.24	-	26.10 (9.84)	14.02 – 52.40	0.57
Currently employed (No)	17 (43)	-	-	17 (43)	-	-	0.99
Rural status (Yes)	13 (33)	-	-	2 (5)	-	-	< .001
Self-reported general health rating	-	3.20 (0.83)	2 – 4	-	2.53 (0.90)	1 – 5	.02
Time since CKD diagnosis [in years]		11.97 (11.08)	0.83 – 36	-	8.17 (6.91)	0.42 – 29	0.20
Time since kidney failure diagnosis [in years]	-	5.49 (7.53)	0.42 – 27	-	3.61 (2.86)	0.08 – 12	0.30
Time since dialysis initiation [in years]	-	1.91 (1.65)	0.08 – 6	-	3.6 (2.87)	0.04 – 12	0.03
Diabetes	7 (37)	-	-	12 (63)	-	-	0.11
Hypertension	16 (47)	-	-	18 (53)	-	-	0.38

*Note*: *0.83 years* = *10 months; 0.42 years* = *5 months; 0.08 years* = *1 month; 0.04 years* = *2 weeks; The Self-reported General Health Rating scale is from 1 (“Poor”) to 5 (“Excellent”)*

### Qualitative findings

**Fig. 1:**
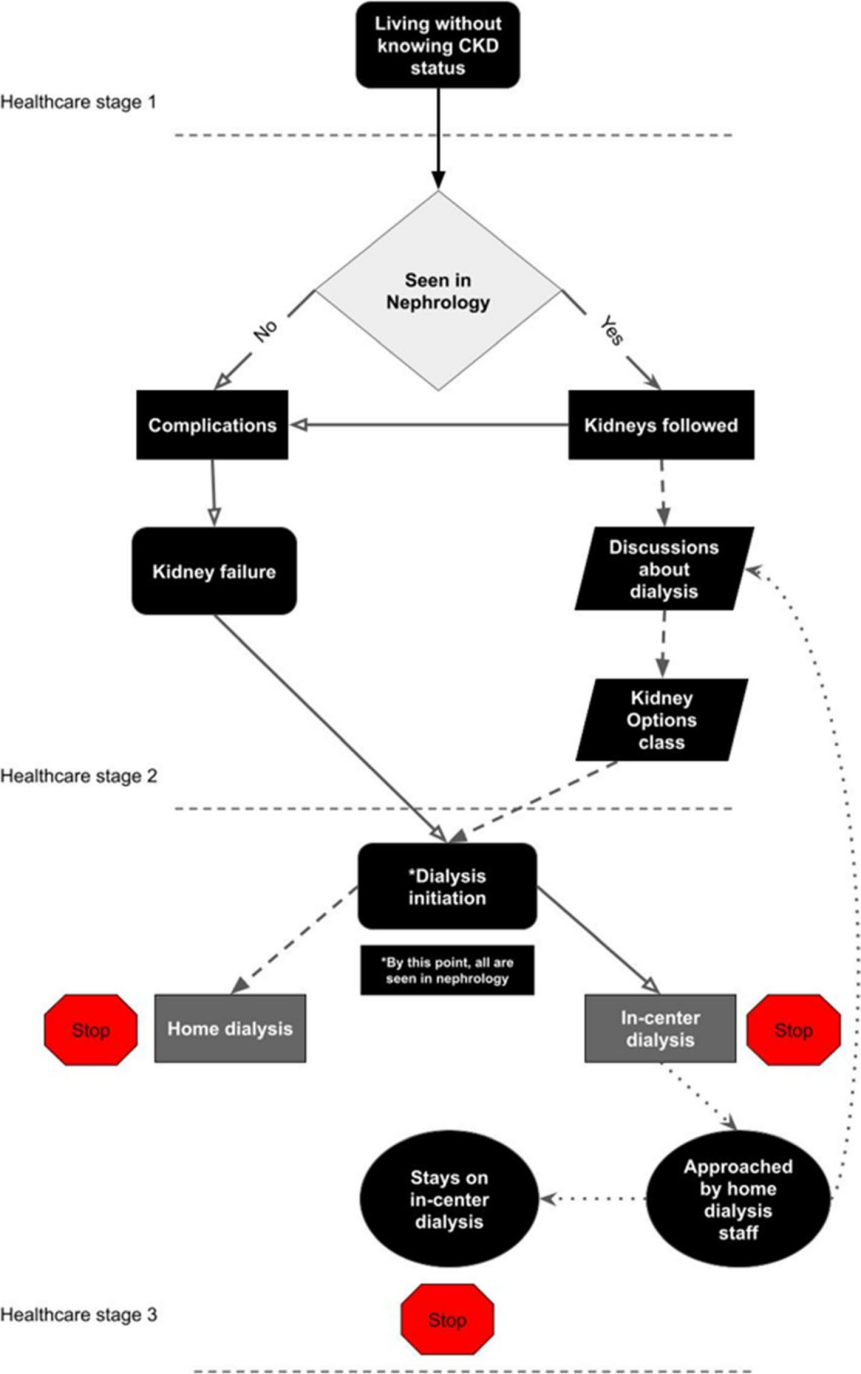
Flow chart to dialysis selection. Three healthcare stages are shown. Similar arrowheads and lines reflect similar trajectories. STOP signs indicate endpoints in participants’ journeys

**Fig. 2:**
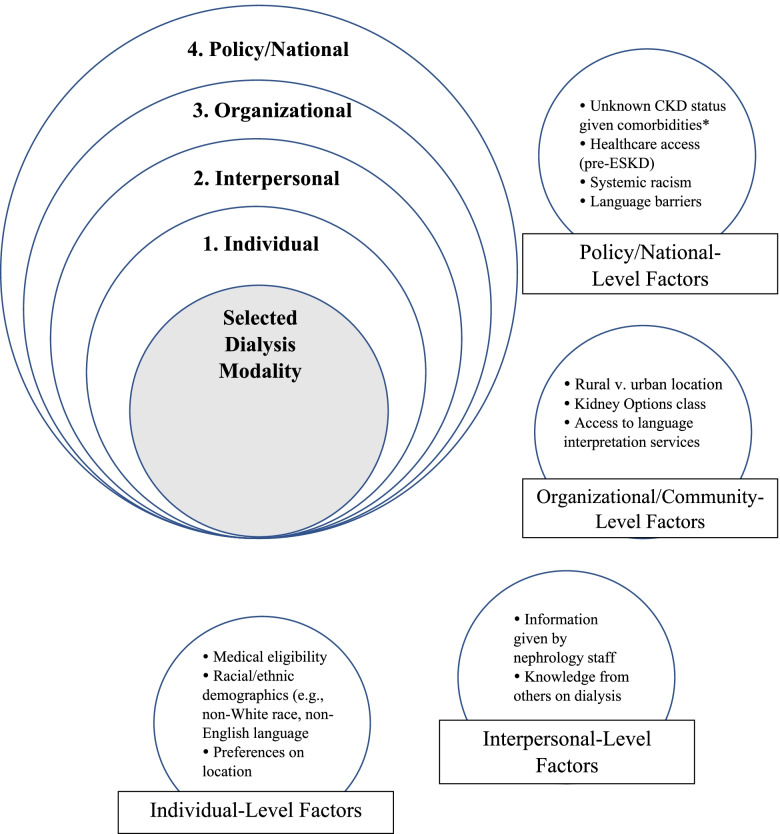
Study findings embedded in the Social Ecological Framework. The figure depicts how the findings are interrelated within a social-ecological system. *Note*: “Unknown CKD status” under Policy/National-level factors refers to unknown CKD status due to lack of screening criteria for patients with common CKD comorbidities*

Six themes emerged following analysis, and there were two different classes of themes. The first three themes refer to three healthcare stages throughout patients’ progression towards ESKD and following dialysis initiation, and describe the different trajectories within these healthcare experiences. Patients’ varied healthcare journeys, culminating to their final dialysis modality, are illustrated in Fig. 1, and depict what each patients’ healthcare journeys looked like without examining details. The final three emergent themes were factors brought up by participants unprompted, and were not previously considered as potentially related to the dialysis decision-making process and the final dialysis modality outcome. These themes describe varying factors that influenced patients’ healthcare experiences leading up to dialysis initiation and thereafter. Quotations below were selected that best illustrate study participants’ experiences. See eTable1 for direct quotations of all themes and subthemes identified from each interview. Findings from this study are illustrated within the SEF in Fig. 2.

### Themes by healthcare stage

#### Living without knowing CKD status

Before becoming aware of their CKD status, participants either actively managed chronic comorbidities within a primary care setting, struggled with healthcare access (e.g., “…they canceled my insurance and then I didn’t get my blood pressure meds…” [In-center]), or elected not to pursue healthcare (e.g., “I was hardheaded. And wouldn’t come to the doctor” [In-center]). Although every patients’ level of healthcare access and healthcare engagement varied, most seemed to understand that managing their own chronic conditions was a priority, even while being unaware they had a diagnosis for CKD:

“Like I said, I've been a diabetic for 15 years. It was very manageable, but they don't tell you about all the health issues that are going to come later.” (In-center)

Overall, greater healthcare access and engagement seemingly corresponded with earlier entry into nephrology care, while less access and engagement seemingly delayed it. Earlier entry into pre-ESKD nephrology care due to greater pre-nephrology healthcare access and engagement seemed commonly reported among participants undergoing home dialysis. For example:

“I would go in for annual physicals and…that’s how he determined…from the labs that this is not going in the right direction, [I] need to go over to see [the nephrologist]” (Home)

versus:

“…the free clinic told me that there was nothing else they can do for me because I had…kidney failure” (In-center)

#### Pre-ESKD nephrology care

Among patients who received pre-ESKD nephrology care, outcomes differed based on them knowing whether dialysis was in their future, and their awareness of other dialysis modalities. For example:

“[I started dialysis] five, six years back. I'm a diabetic… I was going to a kidney

specialist [for six or seven months]. [They were] telling me that the number was going

down, down, down, and that I was going to be in kidney failure… And then that

happened and I had to quit my job and I'm on dialysis.” (In-center).

versus:

“When we first started talking about [home] dialysis, I had read up on it… thought about

the benefits of doing it at home. More convenient.” (Home).

If patients received pre-ESKD nephrology care, they were updated on their progression toward ESKD, and had opportunities to learn about the different dialysis modalities. Information regarding dialysis was provided to patients via nephrology providers and the Kidney Options class hosted by the study site’s nephrology clinic. The Kidney Options class educates patients on all renal replacement therapies offered by the clinic. Many patients attended the Kidney Options class if they dropped below a clinical threshold in kidney functioning; however, the patients who were in between appointments as they reached ESKD automatically initiated in-center hemodialysis. Similarly, those who were not referred to nephrology care prior to reaching ESKD also automatically initiated in-center hemodialysis.

#### Following dialysis initiation: A chance to switch modalities

Patients not medically eligible for home dialysis remained on in-center dialysis, whereas the rest got approached by home dialysis providers while in clinic. This resulted in one of three outcomes: 1) patients attended the Kidney Options class then switched to home dialysis (e.g. “When I went to the meeting for the transplant, they were telling us…you could…do the home or the hemo or another one at home”), 2) patients attended the Kidney Options class but remained on in-center dialysis (e.g. “…even…down the line after you start here you still have that option if you want to… start at home…I chose clinic”), or 3) patients declined to learn more about their options and ultimately remain on in-center dialysis (e.g. “…my doctor brings it up…I don’t want to stab myself…I…don’t want to sleep with a tube…I’d rather be here than do it at home”). Most patients undergoing home dialysis remained on home dialysis provided emergencies did not occur. Overall, satisfaction varied by factors outlined under the emergent themes, and not by modality.

### Emergent themes

#### Dialysis modality “decision-making” v. “selection”

Responses suggest that most patients adopted a passive approach during the decision-making process, in which they pursued the dialysis modality most suitable for them as recommended by nephrology providers regardless of modality. For example:

“[The nephrologist] said it would be best if I would do home dialysis…So, that settled it. The doctor told me, so that was it”. (Home)

“…they really wanted to watch me, especially with my diabetes and stuff like that. If I’m at home, I might get too comfortable.” (In-center)

Even among patients who made the switch from in-center dialysis to home dialysis, the decision-making process was still driven by nephrology providers:

“And then after a couple of years of that hemodialysis in the second round, [the nephrologist] talked to me about the possibilities going on peritoneal dialysis and…we made that switch” (Home)

Only among patients undergoing home dialysis did a few engage in a more proactive decision-making process:

“…got an appointment with [the nephrologist] and…asked him…, ‘Why don't more people do peritoneal’ and [we] decided to go with that…I was concerned [because]…I had some pretty extensive abdominal surgery, but [the nephrologist] said, [they’d] take a shot at it.” (Home)

Some home dialysis patients conducted their own research validating the information given to them, thus improving their comfort level with their dialysis modality (e.g. “…when they gave me information about it, I researched it…And I said, ‘I think this would be better for my life’” [Home]). Having relatives in the medical field, being in the medical field themselves, or knowing someone else on dialysis all supported patients’ basic understanding of dialysis:

“…my dad used to be on [peritoneal]…and my mother…is a nurse so… When I brought it to her she said, ‘Well, your dad did it and he was fine.’” (Home)

However, the amount, accuracy, and desire for knowledge about dialysis varied widely among the sample. In general, limited conversations with nephrology providers before and after dialysis initiation resulted in misinformation regarding dialysis options, primarily among in-center patients. Common misconceptions included believing home dialysis is reserved for emergencies or thinking home dialysis required setting oneself up for hemodialysis.

#### Race, ethnicity, and language

While knowing someone else undergoing dialysis before their own dialysis initiation seemingly put patients in a better position to learn about home dialysis, most Black or Latine patients were undergoing dialysis in-center despite previous familiarity with dialysis (e.g. “When I was a child, I knew what dialysis was. Go get some blood taken out…I just knew when people say dialysis, you think of the big…Puffy arms and hands”). In fact, while 53% of the total sample was White, Blacks and Latine made up 70% of the in-center patient sample. Further, Black and Latine patients reported more interpersonal and structural barriers to healthcare leading up to and following dialysis initiation, including the examples below:

“…the day I got out from my [previous] transplant, I didn't even have coverage for…the anti-rejection medicine. Why wasn't it set up for me…I don't know” (Home)

“My doctor…told me that they had diagnosed me with kidney nephritis…but anyway, it wasn't something that he felt that he should keep up on…I was going to the doctor regularly. How all of that was missed, I don't know.” (In-center)

Primarily Spanish-speaking participants lacked access to dialysis information in Spanish, which led to a poorer understanding of their options. After being asked if someone came to discuss dialysis options with them, this patient explained:

[English-translated] “…they wanted me to sign…I told them, ‘I’m not going to sign anything until you bring me a person, a translator,’ because it was pure English…they said, ‘Ok, I’ll come back later.’ They never came back” (In-center)

Only one participant, a primarily Spanish-speaking patient, indicated a change in their experiences as a dialysis patient following the start of the COVID-19 global pandemic. They discussed how banning guests to limit exposure to potential coronavirus in the hospital impacted their ability to understand nephrology providers:

[English-translated] “[My daughter] used to translate for me…But since COVID they don’t let her come in anymore. Just to drop me off or pick me up…so I don’t have anyone to translate for me.” (In-center)

#### Within-sample quality-of-life & dialysis satisfaction markers

Several participants reported satisfaction with their dialysis when asked about it by the interviewer; however, some responses seemed more enthusiastic than others. For example:

“I’m not trying to put it down. Because it’s making me live” (Home).

versus:

“I much preferred [home]…Because my days are free, when I get up in the morning I'm done with dialysis, and I'm free to do what I want during the day” (Home).

Overall, satisfaction did not seem to vary by dialysis modality. Rather, three factors that emerged when discussing patient satisfaction included fatigue severity, dialyzing schedules, and dialysis access sites. Fatigue severity contributed most to dissatisfaction since some patients experienced either a worsening or an improvement in fatigue following dialysis initiation or a modality switch. For example, if fatigue improved following dialysis initiation or a switch in modalities, they credited the dialysis modality they were undergoing (e.g. “I feel worse when I come, and I feel better when I leave. I feel like, hey, let’s go party, you know?” [In-center]). Other patients reported dissatisfaction regarding their dialyzing schedule, or fistula or catheter access sites.

## Discussion

This qualitative study describes and compares the experiences, knowledge, and attitudes of forty participants undergoing home and in-center dialysis, and aimed to identify macro-level factors associated with dialysis decision-making. Healthcare access and engagement before entering nephrology care, after entering nephrology care, and following dialysis initiation influenced patients’ awareness regarding their CKD status, progression toward ESKD, and dialysis options. Interactions with nephrology providers seemingly influenced awareness the most, but the totality of these experiences affected which dialysis modality patients would eventually undergo. Potential modifiers of these outcomes include race, ethnicity, and language barriers. Most participants adopted a passive role in the decision-making process, following through with the dialysis modality suggested to them by nephrology providers. Finally, fatigue, concerns regarding one’s dialyzing schedule, and problems with fistula/catheter access sites contributed to overall satisfaction with one’s dialysis modality. Because these findings highlighted both micro- and macro-level factors associated with the final dialysis modality (Fig. 2), the SEF seems useful for understanding this phenomenon.

Data-driven analysis identified statistically significant within-sample findings using Pearson’s correlation and Chi-square analysis. The home dialysis cohort were more likely to be White, rural, in a relationship, have more years of education, rate their health as better, and initiate dialysis more recently compared to the in-center cohort. These results, however, should only be interpreted in terms of this small, exploratory study. For instance, home dialysis rates in other clinics may not have rural-majority patient samples; thus, this finding may not be generalizable. On the other hand, the fact that patients undergoing home dialysis were more likely to be in a relationship compared to those undergoing in-center dialysis may suggest that being in a relationship implicitly contributes to one’s self-efficacy to independently manage dialysis at home, even though that finding did not emerge from qualitative interviews. Further research is warranted.

The study sample came from a well-resourced Midwestern hospital with an established home dialysis program. This makes the within-sample findings unique, since the nephrology clinic was equipped with a home dialysis team that provided pre-dialysis education and training for home dialysis use. Thus, the present sample theoretically had the resources to learn about dialysis options prior to and following dialysis initiation. Given the availability of these resources, these findings show that healthcare access and engagement before and after entering nephrology care played a critical role in what dialysis modality patients would eventually undergo.

Findings from the present study are consistent with other qualitative studies outside of the US. One study from Canada (*N* = 12) that interviewed patients undergoing in-center hemodialysis (*n* = 4), home hemodialysis (*n* = 4), and PD (*n* = 4) discussed the difficulty in reaching a quality decision when faced with a limited timeframe [33]. Another study from the Netherlands that interviewed thirteen participants who followed through with a dialysis decision described the important role of nephrology providers in the provision of dialysis information, training, and support when initiating home-based dialysis methods [34]. A qualitative study describing the shared decision-making experiences among patients undergoing in-center dialysis in Australia (*N* = 35) found that participants expressed passivity during their involvement with healthcare-related decisions. Further, primarily Arabic-speaking participants seemed to experience some difficulties with quality decision-making due to language barriers [35]. In conjunction with the present study, these findings suggest there is a need to ensure patients undergoing dialysis are experiencing high quality decision-making even if their approach is passive. Patients who experience communication barriers due to language differences may require greater language interpretation support from professionals in healthcare settings.

Race and ethnicity emerged as a category associated with dialysis modality and differing healthcare experiences. White and non-Hispanic patients made up 75% of the present home dialysis sample but only 30% of the in-center dialysis sample. Based on the qualitative responses, Black and Latine participants experienced less preventive general and kidney-related healthcare compared to White and non-Hispanic participants. Considering the organizational support available at this clinic, these results allude to barriers associated with healthcare access and engagement among patients with CKD who are Black and Latine, and broadly, racial/ethnic inequities in healthcare [36–41]. Although reflective of a small sample, these findings are consistent with national trends. Among national incident dialysis cases, 12.04% of White patients utilized home dialysis, versus 9.56% of Black patients, and 11.79% of non-Hispanic patients, versus 10.60% of Hispanic patients. Among prevalent dialysis cases, 14.08% of White patients utilize home dialysis versus 9.29% of Black patients, and 13.07% of non-Hispanic patients versus 9.90% of Hispanic patients [1]. All five Spanish-speaking patients of limited English proficiency were undergoing in-center dialysis (25% of in-center study sample), which hints at potential obstacles getting patients with limited English proficiency interested in home dialysis. This finding is consistent with other studies identifying language barriers within kidney care, and a broad lack of translation and interpretation support in healthcare [42, 43].

### Limitations

Given the cross-sectional, exploratory nature of this study, causal statements cannot be made, but relationships between factors may be suggested. Because the study required participants to reflect on their experiences retrospectively, their responses were susceptible to recall bias. Although one of the qualitative findings describe the patient approach to decision-making as passive, the interviewer was not present during any patient-provider conversations and could only draw conclusions based on participant descriptions and impressions of the decision-making process. Therefore, these findings should be interpreted with caution. Not every eligible patient within the clinic was interviewed, however, the demographic characteristics of those who declined were similar to the present sample. Some patients within the in-center dialysis clinic declined participation because they felt too tired to participate in an interview while they were dialyzing, so their perspectives were not reflected in the findings. Furthermore, only two participants undergoing home hemodialysis were interviewed. While this low rate included in the sample is consistent with national trends [1], and their responses were consistent with the rest of the home dialysis group, it may be worthwhile for future qualitative studies to confirm whether patients undergoing home hemodialysis and PD have comparable quality of life and decision-making experiences.

All participants came from a tertiary care research hospital in a Midwestern, largely rural US state, where they had access to dialysis education via home dialysis providers and the Kidney Options class offered by the nephrology clinic, even though many dialysis clinics in the US do not offer home dialysis nor do they offer dialysis education to their patients [44]. Therefore, findings may not generalize to other US patients undergoing dialysis. However, these results may be used to generate hypotheses and quantify these relationships in larger samples of patients within cross-sectional, longitudinal and/or multi-site studies.

### Clinical implications

Overall, these findings lay a foundation for future directions for improving patient outcomes in nephrology care. Participant responses suggested that healthcare access affected one’s future modality selection, and furthermore, healthcare access during pre-nephrology care, pre-ESKD nephrology care, and following dialysis initiation were potentially equally influential. Although past interventions have prioritized pre-ESKD nephrology care as the setting for dialysis education to occur [11, 13, 15–18], these findings suggest that broadening the timeframe of messaging surrounding dialysis options to include the other healthcare stages (e.g., Fig. 1) may be effective in increasing home dialysis selection. Post-dialysis initiation may be a particularly pivotal stage because patients can better evaluate whether their fatigue levels, fistula/catheter access sites, and dialyzing schedules are compatible with their lifestyles, and be better equipped to engage more proactively during shared decision-making processes. Providing dialysis information throughout other stages of healthcare may improve patient agency in decision-making, since urgency will be lower as it will not be occurring just before ESKD is reached, thus giving patients more time to weigh their preferences alongside nephrology providers’ recommendations. Special consideration and additional resources should be prioritized for patients of underrepresented races and ethnicities, particularly within nephrology care, since they may have faced greater healthcare challenges leading up to dialysis initiation.

## Conclusions and Public health implications

These results provide important insights into factors associated with dialysis selection beyond patient knowledge and efforts from nephrology providers. Findings that pointed to broader factors affecting dialysis modality selection, including healthcare access and racial/ethnic inequities, seemed to also influence patient knowledge and efforts from nephrology providers. This suggests the existence of a relationship between micro- and macro-level factors surrounding dialysis modality decision-making, which supports modeling this phenomenon under the SEF (e.g., Fig. 2). Framing the phenomenon as such may help identify targets for future studies and interventions. Based on these conclusions, interventions should ideally target macro-level factors to achieve greater equity in dialysis outcomes.

## Supplementary Information

**Additional file 1.** 

**Additional file 2.** 

## Data Availability

The datasets generated and analyzed during the current study are not publicly available due to ethical regulations, but can be available from the corresponding author on specific request, if approved by the university for data protection and information security (i.e.,Institutional Review Board) at University of Iowa.

## References

[1] United States Renal Data System. 2020 USRDS Annual Data Report: Epidemiology of Kidney Disease in the United States: End-Stage Renal Disease. Bethesda, MD; 2020. https://adr.usrds.org/2020/end-stage-renal-disease.

[2] Himmelfarb J, Ikizler TA (2010). Hemodialysis. N Engl J Med.

[3] Ellam T, Wilkie M (2015). Peritoneal dialysis. Medicine (Baltimore).

[4] Harwood L, Leitch R (2006). Home dialysis therapies. Nephrol Nurs J.

[5] Weinhandl ED, Liu J, Gilbertson DT, Arneson TJ, Collins AJ (2012). Survival in Daily Home Hemodialysis and Matched Thrice-Weekly In-Center Hemodialysis Patients. J Am Soc Nephrol.

[6] Cassidy BP, Getchell LE, Harwood L, Hemmett J, Moist LM (2018). Barriers to education and shared decision making in the chronic kidney disease population: a narrative review. Can J kidney Heal Dis.

[7] Porteny T, Gonzales KM, Aufort KE, et al. Treatment Decision Making for Older Kidney Patients during COVID-19. Clin J Am Soc Nephrol. 2022. 10.2215/CJN.13241021 PMC9269620 35672037

[8] Verberne WR, Konijn WS, Prantl K (2019). Older patients’ experiences with a shared decision-making process on choosing dialysis or conservative care for advanced chronic kidney disease: a survey study. BMC Nephrol.

[9] Chen N, Lin Y, Liang S, Tung H, Tsay S, Wang T. Conflict when making decisions about dialysis modality. J Clin Nurs. 2017. 10.1111/jocn.13890 28543737

[10] Vélez-Bermúdez M, Christensen AJ, Kinner EM, Roche AI, Fraer M (2019). Exploring the Relationship Between Patient Activation, Treatment Satisfaction, and Decisional Conflict in Patients Approaching End-Stage Renal Disease. Ann Behav Med.

[11] Green JA, Boulware LE (2016). Patient Education and Support During CKD Transitions: When the Possible Becomes Probable. Adv Chronic Kidney Dis.

[12] Harwood L, Clark AM (2012). Understanding health decisions using critical realism: home-dialysis decision-making during chronic kidney disease. Nurs Inq.

[13] Manns BJ, Taub K, VanderStraeten C (2005). The impact of education on chronic kidney disease patients’ plans to initiate dialysis with self-care dialysis: A randomized trial. Kidney Int.

[14] Morton RL, Tong a, Howard K, Snelling P, Webster a C. The views of patients and carers in treatment decision making for chronic kidney disease: systematic review and thematic synthesis of qualitative studies. BMJ. 2010;340(7742):350. 10.1136/bmj.c112 PMC2808468 20085970

[15] Murray MA, Brunier G, Chung JO (2009). A systematic review of factors influencing decision-making in adults living with chronic kidney disease. Patient Educ Couns.

[16] Wuerth DB, Finkelstein SH, Schwetz O, Carey H, Kliger AS, Finkelstein FO (2002). Patients’ descriptions of specific factors leading to modality selection of chronic peritoneal dialysis or hemodialysis. Perit Dial Int.

[17] Harwood L, Clark AM (2013). Understanding pre-dialysis modality decision-making: A meta-synthesis of qualitative studies. Int J Nurs Stud.

[18] Morton RL, Snelling P, Webster AC (2012). Dialysis modality preference of patients with CKD and family caregivers: A discrete-choice study. Am J Kidney Dis.

[19] Bronfenbrenner U. The Ecology of Human Development: Experiments by Nature and Design. Harvard university press; 1979.

[20] Golden SD, Earp JAL (2012). Social Ecological Approaches to Individuals and Their Contexts. Heal Educ Behav.

[21] Stokols D (1996). Translating social ecological theory into guidelines for community health promotion. Am J Heal Promot.

[22] Kvale S, Brinkmann S. Interviews: Learning the Craft of Qualitative Research Interviewing. sage; 2009.

[23] Sofaer S (1999). Qualitative methods: what are they and why use them?. Health Serv Res.

[24] Armat MR, Assarroudi A, Rad M, Sharifi H, Heydari A (2018). Inductive and deductive: Ambiguous labels in qualitative content analysis. Qual Rep.

[25] Tong A, Sainsbury P, Craig J (2007). Consolidated criteria for reporting qualitative research (COREQ): a 32-item checklist for interviews and focus groups. Int J Qual Heal care.

[26] Bradley EH, Curry LA, Ramanadhan S, Rowe L, Nembhard IM, Krumholz HM (2009). Research in action: Using positive deviance to improve quality of health care. Implement Sci.

[27] Palinkas LA, Horwitz SM, Green CA, Wisdom JP, Duan N, Hoagwood K (2015). Purposeful sampling for qualitative data collection and analysis in mixed method implementation research. Adm Policy Ment Heal Ment Heal Serv Res.

[28] WWAMI RUCA Rural Health Research Center. Rural-Urban Community Area Codes (RUCAs). http://depts.washington.edu/uwruca/ruca-uses.php. Published 2019.

[29] Agriculture USD of. Rural-Urban Commuting Area Codes. https://www.ers.usda.gov/data-products/rural-urban-commuting-area-codes.aspx. Published 2020.

[30] Fusch PI, Ness LR (2015). Are we there yet? Data saturation in qualitative research. Qual Rep.

[31] Saunders B, Sim J, Kingstone T (2018). Saturation in qualitative research: exploring its conceptualization and operationalization. Qual Quant.

[32] Dedoose. Web application for managing, analyzing, and presenting qualitative and mixed methods. 2019.

[33] Cassidy BP, Harwood L, Getchell LE, Smith M, Sibbald SL, Moist LM (2018). Educational support around dialysis modality decision making in patients with chronic kidney disease: qualitative study. Can J kidney Heal Dis.

[34] Finderup J, Jensen JD, Lomborg K (2021). Shared decision-making in dialysis choice has potential to improve self-management in people with kidney disease: A qualitative follow-up study. J Adv Nurs.

[35] Muscat DM, Kanagaratnam R, Shepherd HL, Sud K, McCaffery K, Webster A (2018). Beyond dialysis decisions: a qualitative exploration of decision-making among culturally and linguistically diverse adults with chronic kidney disease on haemodialysis. BMC Nephrol.

[36] Boulware LE, Mohottige D (2021). The seen and the unseen: race and social inequities affecting kidney care. Clin J Am Soc Nephrol.

[37] Cohen RA, Martinez ME, Zammitti EP. Health Insurance Coverage: Early Release of Estimates from the National Health Interview Survey, 2015.; 2016. chrome-extension://efaidnbmnnnibpcajpcglclefindmkaj/viewer.html?pdfurl=https%3A%2F%2Fwww.cdc.gov%2Fnchs%2Fdata%2Fnhis%2Fearlyrelease%2Finsur201605.pdf&clen=679975&chunk=true

[38] Eberly LA, Richterman A, Beckett AG (2019). Identification of racial inequities in access to specialized inpatient heart failure care at an academic medical center. Circ Hear Fail.

[39] Kirby JB, Kaneda T (2010). Unhealthy and uninsured: exploring racial differences in health and health insurance coverage using a life table approach. Demography.

[40] Paredes AZ, Hyer JM, Diaz A, Tsilimigras DI, Pawlik TM (2020). Examining healthcare inequities relative to United States safety net hospitals. Am J Surg.

[41] Yearby R (2011). Racial Inequities in Mortality and Access to Health Care: The Untold Peril of Rationing Health Care in the United States. J Leg Med.

[42] Cervantes L, Rizzolo K, Carr AL (2021). Social and Cultural Challenges in Caring for Latinx Individuals With Kidney Failure in Urban Settings. JAMA Netw open.

[43] Pandey M, Maina G, Amoyaw J, et al. Impacts of English Language Proficiency on Healthcare Access, use, and Outcomes among Immigrants: A Qualitative Study. 2021. 10.1186/s12913-021-06750-4 PMC8314461 34311712

[44] Chan CT, Blankestijn PJ, Dember LM (2019). Dialysis initiation, modality choice, access, and prescription: conclusions from a Kidney Disease: Improving Global Outcomes (KDIGO) Controversies Conference. Kidney Int.

