# Supplementary material for: Disparities in dialysis modality decision-making using a social-ecological lens: a qualitative approach
Source: BMC Nephrol. 2022 Aug 5;23:276. doi: 10.1186/s12882-022-02905-5 (PMC9356453; doi:10.1186/s12882-022-02905-5)
Supplement: Supplementary file 1 — Additional file 1. [file 12882_2022_2905_MOESM1_ESM.docx]

**eTable 1.** Representative quotations for each overarching theme and subtheme.

| **“HEALTHCARE STAGE” THEMES** |
| --- |
| ***Living without knowing CKD Status*** |
| **Comorbidities & complications** |
| You don’t know you’re sick until they tell you, I think. (P8, Female [F], Home dialysis)  I’d been extremely tired, no energy, out of breath…I started seeing a doctor. And…one day, after several tests and stuff, they said that I was having kidney failure. (P12, Male [M], Home dialysis)  …cuz you know my blood sugar always be high. And that's the symptoms of your kidney's going to fail. And that was it...The diabetes you know then kidney failure, and then you go blind, and then you cut your leg off. (P26, F, In-center dialysis)  By the time we were talking to the nephrologist the fate was sealed. (P11, M, Home dialysis)  I mean, there's nothing that you can do. Like I said, I've been a diabetic for 15 years. It was very manageable, but they don't tell you about all the health issues that are going to come later. (P31, M, In-center dialysis) |
| **Healthcare access** |
| So we, my wife and I would go in for annual physicals and uh, and then that’s how he determined you know from the labs that this is not going in the right direction, you need to go over to see [the nephrologist]. (P3, M, Home dialysis)  I didn't even know my kidneys were that bad before all this, they all went bad on me. They were actually still at 50% function and they canceled my insurance and then I didn't get my blood pressure meds that my blood pressure's really high, way over 200. I never had it checked before. That's probably what, they said that's the main reason my kidneys stopped working was super high blood pressure. (P19, M, In-center dialysis)  My doctor…had told me that they had diagnosed me with kidney nephritis… but anyway, it wasn't something that he felt that he should keep up on, and so I moved here in 2010…I was going to the doctor regularly. How all of that was missed, I don't know… (P37, F, In-center dialysis)  So I was going to the free clinic and the free clinic told me that there was nothing else they can do for me because I had a kidney failure. (P40, F, Home dialysis) |
| **Healthcare behaviors** |
| [Original] La primera vez cuando me detectaron la infección, yo fui a una clínica que es gratis, a chequearme, y ellos me mandaron a [otro hospital]…[el otro hospital] me dio unos medicamentos, … ellos me dijeron, "Tienes que ir a la Universidad de la clínica para que ellos te atiendan." Yo no vine, nomás terminé los medicamentos…me descuidé yo, por eso no vine a buscar la información.  [English-translated] The first time when they detected the infection, I went to a free clinic, to get myself checked, and they sent me to [another hospital]…[the other hospital] gave me some medications,…they told me, “You need to go to the University’s clinic so they can take care of you.” I didn’t go, I only finished taking the medications…I was careless with myself, and that’s why I didn’t look up the information. (P30, F, In-center dialysis)  I would say I was hardheaded. And wouldn't come to the doctor and see. I could've been in real serious trouble. Not coming to the hospital and get diagnosed what was wrong with me. (P32, M, In-center dialysis) |
| ***Pre-ESRD Nephrology Care*** |
| **Conversations with nephrology staff about dialysis and/or ESRD** |
| I think the readings were at uh, at about 10% level? … I had come up here to see if I qualified for a cadaver…transplant. And anyhow, this one doctor looked at me, ‘Man, you could run it down to 6.’ So I’m thinking, well I’ve got a ways to go yet. [the nephrologist] says, ‘No. No. You don’t.’ (P3, M, Home dialysis)  Well, they, they told me my kidneys was failing and then they put [the fistula] in my arm…and I went for about a year without it, without anything. But then [my kidneys] crashed…Yeah, you know, I was seeing a doctor, but they was like, I don't know, it was kinda too late for my diet and all that. I don't know they was just waiting for [my kidneys] to crash. (P5, M, In-center dialysis).  [I started dialysis] five, six years back. I'm a diabetic. And I was working, and I was going to a kidney specialist [for six or seven months]. He was telling me that the number was going down, down, down, and that I was going to be in kidney failure. And then that happened and I had to quit my job and I'm on dialysis. (P26, F, In-center dialysis)  [Dialysis] was definitely in my future…my doctor prepared me as much as [they] could. (P27, F, In-center dialysis)  I didn't really understand what dialysis was though. I wish someone would've yelled at me or something…[the doctors] told me something about dialysis, but...I didn't know what it was…When did I find out what is dialysis? When I had to get dialysis. (P33, M, In-center dialysis) |
| **Dialysis knowledge** |
| When we first started talking about dialysis, I had read up on it. My wife had read up on it. Wife’s a nurse, so we both looked into it, and we thought about the benefits of doing it at home. More convenient. (P12, M, Home dialysis)  I knew [dialysis was in my future]. I never expected it to start as young as I did, ok? Because my dad was 60 when he started, ok? I never expected it to be now. That's kind of a two face or double-edged sword. I look at it as disappointed that I started in my 50s? Sure, but on the same token, I feel fortunate, because I'm in pretty good shape. (P21, M, Home dialysis) |
| **Desire for knowledge** |
| I just thought I would be sick when I get older. Didn't realize how it could go bad. Didn't realize that the disease I have is my immune system…Learn everything you can. Take the class, ask all questions, ask the dumbest questions. Yeah. I would just learn. Don't learn while you're in dialysis. Learn before you get there. (P33, M, In-center dialysis) |
| **Healthcare access** |
| I went to the doctor like, um, when I moved here [14 years ago], then I immediately started seeing a doctor here. And um, he told me, a kidney doctor told me like about 2006, he said in about 7 years, you’re gonna start having complications. And … I started with the episode in 2017… that was like my first time I ever seen [a nephrologist]. (P1, F, In-center dialysis)  They didn't [give me good enough health insurance] because I was a teaching assistant, you know? (P37, F, In-center dialysis) |
| ***Following Dialysis Initiation: A chance to switch modalities*** |
| **Conversations with nephrology staff about dialysis and/or ESRD** |
| It was just too much to take. You know your whole life changes within few hours and then it was too much information every day and it was too much information of everything all the time, and then hospital regulations, they have to have your insurance straight and all this. So it was always something. (P20, M, Home dialysis)  …they give you a paper like this here to study. And ask do you still want to learn how to do it at home. A lot of people did learn how. But I didn't want to learn how (P32, M, In-center dialysis)  … the one doctor…says, "You should do home dialysis." It's like, well, I don't want to stab myself. And I also don't want to sleep with a tube. So... I'd rather be here than do it at home. (P33, M, In-center dialysis)  When I went to the meeting for the transplant, they were telling us the different ways. Which way you could go to do it, you do the home or the hemo or another one at home, you could do too. I forgot the name of it. (P14, M, Home dialysis)  Yeah, they gave me the option. You had a option to do whichever you want to do…because once you get to the point of going on dialysis because you to do dialysis, they give you a option there. Then even on down the line after you start here you still have that option if you want to stop here and start at home. You have that option, yeah. (P37, F, In-center dialysis) |
| **Satisfaction with or preferring current modality** |
| In-center would be, if your machine explodes or your house burns down or you’re traveling, in my opinion, that’s what in-center is for, for us. But otherwise, home hemo is the way to go...The dialysis shouldn’t be your gravity, it shouldn’t be what keeps you on the earth. (P11, M, Home dialysis)  Just for the socialization…I had made up my mind that I needed to get out of the house…At least the people understand when I come here. (P27, F, In-center dialysis)  Like I said, I can do it when I need to. Go anytime when I please. I don't have to get out the house and drive, my supplies are delivered to my home. Everything is convenient. And I'm more happier. I know that this is something different new to my life, but at least I can do it in my home. It's private. It's in my bedroom, I don't have to be bothered with anybody and sleep while I do it. Any time of the day I can do it. (P40, F, Home dialysis)  …just having a tube and taking showers like that half-way in or something and worrying about if it's dirty all the time, I'd just rather not. I mean, I don't do a lot of things. So it kind of feels better to come over here. Kind of feels like a job…So, I'd rather be here than do it at home… It's like family. I give them [the staff] gifts sometimes and on certain holidays…So I mean, I like it. It's just the repetitiveness. Instead of being at home, where I would just do nothing. (P33, M, In-center dialysis) |
| **Misinformation and/or lack of knowledge regarding dialysis** |
| [Original] No hay ningún problema. Sí, claro. Por eso te digo, si puedo ir a hacerlo en mi casa estaría mucho mejor.  [English-translated] There’s no problem. Yes, of course. That’s what I’m saying, if I could do it at home that would be much better. (P29, M, In-center dialysis)  You mean poke myself? No…I probably would stay at home if somebody else is going to poke me. (P5, M, In-center dialysis)  [Home dialysis is] like something that, that’s an option when you’ve been on it for a while. The, when they first put me on it was just uh, making sure I stay alive and not to get so far that I was like, you know really in bad shape… It was, but I was just, didn’t have any option, they just told me, ‘That’s where you’re going’… It was just uh, what opened up. There was an opening, cuz there was, you know, to keep me alive. There wasn’t much options about it. (P2, M, In-center dialysis) |
| **Desire for knowledge** |
| Even though I was afraid of getting some of the other needles. Yeah, I really think that they gave more of a presentation about home dialysis. Is that what they want, everyone to do it at home? Is that what, is that their push or? Do you know what I mean? Because it was like there was a lot of information on that than there was on [in-center hemodialysis]…I needed to prepare a little more. It would have been a little more helpful [to have more information about in-center hemodialysis]. (P27, F, In-center dialysis)  Yeah. I wish I would've known that this was dialysis. I have to live off a machine... I wish someone would've warned me instead of saying, "Oh, this could just happen." Said that, "No, hey. You keep going down this road, this is where you're going to be. You're going to be in a chair sitting down with your arm sitting right here all the time. And you're going to go to dialysis every other day... I just wish someone would've shook me. (P33, M, In-center dialysis) |
| **Modality switch** |
| And then after a couple of years of that hemodialysis in the second round, [the nephrologist] talked to me about the possibilities going on peritoneal dialysis and I was interested in that and we made that switch… Yeah, I think I did express a concern about how terrible the hemodialysis made me feel. And [the nephrologist] took that seriously and listened to me very carefully. (P10, M, Home dialysis)  I think I would've done [the switch] sooner. I wouldn't have waited as long as I waited because it wasn't because I wasn't ready. It wasn't because them or they have too many people. It wasn't because of that. It was my decision. They've asked me and they're, "I'll come back next month and I'll ask you again, if you're ready for it." And I was like, "Ok." "And then I'll come back and if you're this..." And I was like, "Ok." And then like every other Monday would ask me, so I wish I would have done it sooner. (P20, M, Home dialysis) |
| **“EMERGENT” THEMES** |
| ***Dialysis Modality “Decision-Making” v. “Selection”*** |
| **Provider-led discussions** |
| …the staff here recommended [PD]. They thought I'd be a good candidate (P21, M, Home dialysis)  Well, again, the options were, “You should do this, you should do this home thing because it's easy and it's quick and it's good.” Um, and I would want the hemo and, uh, it didn't have a real, uh, choice in that either because I got on that because a catheter was healing on the other. Um, I don't know. I just feel like, uh, I probably, wouldn't just, if this is gonna be the way it has to go I just never questioned it… I would like probably more time making a choice or discussing the options. Um, I did, I did after the first, uh, well, while I was in training with the peritoneal, I did ask to have a different doctor, because I thought the one doctor wasn't, [they] scared me a little, pushy, and they did give me the other [doctor], which made me very at ease, so I mean they, uh, the university did, uh, I don't want to cater, but did listen to my choices. (P22, M, Home dialysis)  Well, initially, I thought I would do the fistula and the hemo…Because it said you'd do it for three or four hours and three or four times a week. And I thought, well, heck I sit around for three or four hours a day. That's not a problem. And then I had a conversation with [nephrology nurse] and she talked me into PD. (P24, M, Home dialysis)  Because they really wanted to watch me, especially with my diabetes and stuff like that. If I'm at home, I might get too comfortable. I can do it whenever. (P31, M, In-center dialysis)  Well, [the nephrologist] said it would be best if I would do home dialysis, because it would be better on your system, and you're a little person. So really you should do home dialysis. So, that settled it…The doctor told me, so that was it. That was why. (P39, F, Home dialysis)  Well, after I had the classes, [I had enough information]. Ok. I did not before. So they're very nice about telling you exactly, and doing it here several times. So, those teachers are good. So, that's how I learned…They tell you everything here. They told me everything. Yeah. And they wouldn't let you go home unless you knew it all. So you feel comfortable…They told me all the answers here. You know, they were really nice about it. I knew nothing. I knew nothing. (P39, F, Home dialysis) |
| **Perceived options or choices** |
| I was given the option of PD because I think kidneys were higher functioning, because I still produce quite a bit of residual urine, and I'm sure part of it is because of [my husband]. (P6, F, Home dialysis)  Really wasn’t that hard to make the decisions on your own. And get somebody to listen. That’s where it gets hard. (P8, F, Home dialysis)  Well, they said I could possibly do it at home or come in every day. I'd rather do it this way. They said that takes like eight hours a day, every day. That's kind of crazy too. (P19, M, In-center dialysis)  I don't think anybody took my situation into account…for me, the easiest thing would be go in for the hemodialysis to the [closer] site and set up, you know, it's only two blocks from my house. So that would be the best choice for me. (P22, M, Home dialysis) |
| **Lack of discussion with nephrology staff** |
| [Original] E: ¿Te hablaron de esas opciones o no?  P: No, me parece que no.  I: ¿Tú sabías que existían esas opciones?  P: La mera verdad casi no, no sabía tanto que podía hacer eso.  I: ¿Te interesa hacerlo [en casa] o no?  P: Claro. Si hubiera eso, está mucho mejor para mí.  I: ¿Por qué sería mejor?  P: No tendría que estar viniendo, mi hija a traerme y volverme a recoger. Lo otro sería de que cuando hay nieve ella tiene que manejar. A veces no duerme bien y eso podría ser un poquito difícil.  [English-translated]  I: Did they talk to you about those options or no?  P: No, seems to me they didn’t.  I: Did you know those options existed?  P: The simple truth is not really, I didn’t really know that I could do that.  I: Does it interest you to do it at home, or no?  P: Of course. If that existed, it’d be much better for me.  I: Why would it be better?  P: I wouldn’t need to keep coming here, my daughter brings me and picks me up. The other thing is when there’s snow she has to drive. Sometimes she doesn’t sleep well and it could be a little difficult.  (P29, M, In-center dialysis)  I'm surprised they haven't said that [home hemodialysis is an option] because they keep saying, are you on the waiting list in [other dialysis clinic]? I didn’t know there were other options for that, but…I've got plenty of support [at home] if I needed it. (P22, M, Home dialysis)  [Original] Sí sabía [lo que era diálisis], pero no entendía cómo se hacían. Necesitaba más detalles y todo.  [English-translated] Yes I knew what dialysis was, but I didn’t understand how it was done. I needed more details and stuff. (P35, M, In-center dialysis) |
| **Desire for knowledge regarding dialysis** |
| …talk to other people that are honest before making a choice, both sides of the hemo and the other. And I, I think one of the things, questions I still have, what's the long-term effects to both of those types of procedures. I mean, when you're on peritoneal, uh, will you ever get off of that and have to go to a game or vice versa? And then is there ever a time that you can get off of it totally without having a transplant? … I think the, the interaction, the one-on-one contact with others [to learn about dialysis] would have been, for me the reading part, uh, one, uh, my eyesight's a little bad, so it’s difficult. And just, just hearing the experiences of other people with it would be nice. [Interviewer: What about like a video format?] Again, yeah, but still there's, they're telling you what's going on. You don't have the option to ask questions. Give me your questions. (P22, M, Home dialysis)  I wish I would have had more time of understanding everything. It was just a lot of having to get on the phone, call my sister…That's what you have to do…you definitely don't want anything getting worse. (P31, M, In-center dialysis) |
| **Information-seeking behaviors** |
| I looked [the options] up myself…I went on, and I looked to see, and then I heard PD. And I'm like, ‘Ooh, I like that.’…[Got information from] Library, and friends, I know they all worked at the hospital, friends were talking about it. (P8, F, Home dialysis)  And got an appointment with [the nephrologist] and just talk to him and kind of asked him like, you know, “Why don't more people do peritoneal” and this and that and decided to go with that… I was concerned with, because one of the things that I had read, if you have a lot of scar tissue in your abdomen, you might not be a candidate. And I had some pretty extensive abdominal surgery, but he said, he'd take a shot at it. (P23, F, Home dialysis)  I knew I wanted to do home dialysis. I didn't really know a lot about peritoneal… I was kind of investigating a little bit more about this. It's like, “Oh, this sounds like it'll fit my lifestyle way better.” (P23, F, Home dialysis)  …they give you options if you want to get a fistula, so I just think it was... I don't even remember who told me about peritoneal, but when they gave me information about it, I researched it and looked at it. And I said, "I think this would be better for my life" … I researched it online and then they gave me pamphlets on it. I still have them at home. (P40, F, Home dialysis) |
| **Knowing someone else on dialysis & medical field insight** |
| My son has…been on peritoneal dialysis [and] hemodialysis. So he’s done everything. He’s been there, he’s done it. And uh so I called him and I asked him, he said, “Dad” he said, “with your lifestyle you need to be on peritoneal dialysis” because I like to be able to do whatever I want to do every day of the week, I don’t wanna be tied up 3 days of the week. (P3, M, Home dialysis)  I have a friend who did do hemodialysis and he said that uh, he was very tired after doing it, you know. You could go in the morning, but he said he was tired the rest of the day and it just wore him out. I don’t know, I’ve never been through it so I don’t understand that… Doing this? Doing peritoneal? Well I’m sleeping through it so it doesn’t wear me out. (P3, M, Home dialysis)    Um, [driving a half hour] would be [a long time] three times a week. And then, I mean, it's actually an hour, both ways. And I know sometimes there was a lady … that did it and she was so tired. She couldn't drive herself. So then she had to get a driver. So then they would drive over and then drive back and then have to drive over and pick her up and drive back. And yeah, that's inconvenient. I get to crawl in my comfy bed and hook up my tube and yeah. (P23, F, Home dialysis)  [My wife] was supportive. She was definitely up for it. We're empty nesters. And she's a nurse practitioner... She's got the basic medical background that all nurses has. She thought that the PD was a good idea and she wanted to get things going and she's very helpful. Now, I've gotten my weight, my lifting restrictions lifted from my surgery, but she's been loading my bags and dumping it in the morning and as long as she is able to keep doing it, I'm going to let her. (P24, M, Home dialysis)  …my dad used to be on it. Yeah, so my dad did peritoneal and my mother had told me about it. Yeah. When I brought it to her she said, "Well, your dad did it and he was fine”…My mother is a nurse so she just was on my side and supportive because the thing with that she just told me I had to... She didn't tell me to do it. She just talked to me about it, but she was really cautious because she said it had to be up to me. She said that when you make the choice to have that done, you wanted it. You wanted peritoneal and she just supported me because she didn't want to tell me to just go do this and have a surgery. She's not like that. So she was very supportive. And then after we've had the surgery, she was very pleased and happy that I chose. (P40, F, Home dialysis) |

| ***Modifiers: Race, ethnicity, and language*** |
| --- |
| **Responses from Black and Latine patients** |
| *Experiences in nephrology* |
| Yeah. A couple of nurses telling me about [home dialysis]. Some were thorough with it, some doctors didn’t ... and just ... Because they really wanted to watch me, especially with my diabetes and stuff like that. If I’m at home, I might get too comfortable. I can do it whenever. [Interviewer: Do you think that you’d get too comfortable?] No, not really…But they was just basically telling me there’s a lot of people here that’s outpatient, inpatient. I’m not saying that they would say, “Hey, we need the space. So we just want you to do home dialysis.” It was just the options. (P31, M, In-center dialysis)  I don’t understand because when I got sick in 2017, they did the dialysis, but after that, after I left the hospital, I didn’t have to have it no more. And then all of a sudden my doctor ran some tests and said they think go ahead with the dialysis and that was only around Christmas time, whatta Christmas present because that’s when I was told that I needed to start it that next week. (P1, F, In-center dialysis)​  [Original] E: ¿Te hablaron de esas opciones o no?  P: No, me parece que no.  I: ¿Tú sabías que existían esas opciones?  P: La mera verdad casi no, no sabía tanto que podía hacer [diálisis en el hogar].  I: ¿Te interesa hacerlo [en casa] o no?  P: Claro. Si hubiera eso, está mucho mejor para mí.  [English-translated]  I: Did they talk to you about those options or no?  P: No, seems to me they didn’t.  I: Did you know those options existed?  P: The simple truth is not really, I didn’t really know that I could do that.  I: Does it interest you to do it at home] or no?  P: Of course. If that existed, it’d be much better for me.  (P29, M, In-center dialysis) |
| *Healthcare access* |
| What’s real interesting with that, the day I got out from my [previous] transplant, I didn’t even have coverage for the medicine I needed, the anti-rejection medicine. Why and why wasn’t it set up for me to have, I don’t know, but I got medicine through the Kidney Foundation until the social workers worked out for me to even get my medicine. Yeah. That was a scary feeling too. (P14, M, Home dialysis)  I just moved here and they wouldn’t take me, they tried to get me to move back to [my home city]. They didn’t have a chair [in the in-center clinic] for me. So what happened was I went through the emergency room every day until they, how shall I say, until they found a chair for me… I had to go through the months around, wait on an extra dialysis nurse, trying to come get me and do the treatment. And that could be four or five hours or whatever wait for somebody to even do it. (P14, M, Home dialysis)  My doctor…had told me that they had diagnosed me with kidney nephritis… but anyway, it wasn’t something that he felt that he should keep up on…I was going to the doctor regularly. How all of that was missed, I don’t know…if you’re working you don’t have insurance, so you don’t go to the doctor as regularly because you don’t have none of the paperwork, you know? They put you in a rock and a hard place with it. (P37, F, In-center dialysis) |
| *Knowing someone on dialysis* |
| When I was a child, I knew what dialysis was. Go get some blood taken out. But I didn’t know the process. I think, “Oh, it’s nothing”… I just knew when people say dialysis, you think of the big arm. Puffy arms and hands and stuff like that… I always seen my mom, friends and stuff like that and other patient being on dialysis and yeah. I never even thought of myself being on dialysis. Doctors always have told me you’re going to end up on dialysis. And, just telling a kid that at a young age is very scary. (P31, M, In-center dialysis)  [Original] En México hay muchos [que están haciendo diálisis]. Conoce a un amigo que también lo está haciendo.  [English-translated] In Mexico there are many that are doing dialysis. I know a friend that’s doing it as well. (P28, F, In-center dialysis) |
| **Responses from White patients for comparison** |
| *Experiences in nephrology* |
| I think the readings were at uh, at about 10% level? … I had come up here to see if I qualified for a cadaver uh, transfer and uh, transplant not transfer. And anyhow, this one doctor looked at me, ‘Man, you could run it down to 6.’ So I’m thinking, well I’ve got a ways to go yet. Dr. [Name of nephrologist withheld] says, ‘No. No. You don’t.’ (P3, M, Home dialysis)  But the nursing staff, the dialysis nurses are absolutely tremendous. And that entire unit at the hospital is a blue ribbon group and they're fantastic. And so I was given education about dialysis from them and I underwent training on how to use the machines through them and so forth. And they have a counselor on staff and dietician and who all of whom are tremendous. I mean they were doing a terrific job and so they gave me pamphlets and reading material as well. I had everything I needed from them. (P10, M, Home dialysis)  And then after a couple of years of that hemodialysis in the second round, [the nephrologist] talked to me about the possibilities going on peritoneal dialysis and I was interested in that and we made that switch. (P10, M, Home dialysis) |
| *Healthcare access* |
| The hospital … [referred me] because I lived there for a while and then I moved back here closer to my family…[the nephrologist] was telling me that [kidney failure] was getting closer. (P4, F, Home dialysis)  So we, my wife and I would go in for annual physicals and uh, and then that’s how he determined you know from the labs that this is not going in the right direction, you need to go over to see [the nephrologist] (P9, M, Home dialysis) |
| *Knowing someone on dialysis* |
| My son has had three kidney transplants and in between transplants, he’s been on peritoneal dialysis or hemodialysis. So he’s done everything. He’s been there, he’s done it. And uh so I called him and I asked him, he said, “Dad” he said, “with your lifestyle you need to be on peritoneal dialysis” because I like to be able to do whatever I want to do every day of the week, I don’t wanna be tied up 3 days of the week. (P3, M, Home dialysis)  My father had kidney disease, and so then we started tracking all of the family siblings and stuff. It was well over 20. I can't give you the exact date, but here at the [nephrology clinic], they started tracking me. And I started actually dialysis [in] '18 (P21, M, Home dialysis) |
| **Language barriers** |
| [Original] Lo que pasa es que mi hija sabe [inglés]. Ella me traía, para todo ella viene. Ella me traducía y todo eso. Lo que pasa es [desde COVID] que no la dejan entrar a ella. Nada más para venir a dejarme o para venirme a traer… entonces no hay quién me traduzca...  [English-translated] The thing is my daughter knows [English]. She would bring me, she would come for everything. She used to translate for me and all that. But since COVID they don’t let her come in anymore. Just to drop me off or pick me up…so I don’t have anyone to translate for me. (P29, M, In-center dialysis)  [Original] …querían que yo firmara. Me acuerdo que fue una…y quería que yo le firmara los papeles. Yo le dije, "Yo no te voy a firmar nada si tú no me traes una persona, una traductora", porque era puro inglés. Yo no quería firmar nada, porque yo no entiendo. Ella no hablaba español, la persona que fue y ella quería que yo le firmara los papeles…Le digo, "No te puedo firmar, lo siento, yo no te lo puedo firmar ese papel", no lo firmé y se fue. Dijo, "Ok, regreso al rato". Nunca regresó.  [English-translated] …they wanted me to sign. I remember that it was a…and she wanted me to sign the papers. I told her, “I’m not going to sign anything for you until you bring me a person, a translator,” because it was pure English. I didn’t want to sign anything, because I don’t understand. She didn’t speak Spanish, the person that came and she wanted me to sign the papers. I told her, “I cannot sign it, I’m sorry, I cannot sign that paper,” I didn’t sign it and she left. She said, “Ok, I’ll return later.” She never returned. (P30, F, In-center dialysis) |
| ***Within-Sample Quality-Of-Life & Dialysis Satisfaction Markers*** |
| **Fatigue** |
| One girl said that, “We didn’t know you was gonna be here that long.” She said because I was sick, she said, “You was looking sick,” I would poop on myself. She said, “You was looking real bad,” she said, now you know I’m so perked up when I come in. I say, “Hey everybody!” You know I’m speaking to everybody when I come in, I sit here, I joke with everybody. At first I wasn’t doing that. (P1, F, In-center dialysis)  I sleep better because I’m not being woken up by the drain pains. (P11, M, Home dialysis)  …plus the other dialysis is really exhausting. So this one, it's a little bit more calm, gentle with your body. So I mean, you do it while you're just sleeping. (P20, M, Home dialysis)  I've talked to several people in here about how they feel. I feel better. I feel worse when I come, and I feel better when I leave. I feel like, hey, let's go party, you know? Seriously good. Like I'd rather, yeah I feel better. I mean, I actually had them push me up in the wheelchair, but I walk out. (P27, F, In-center dialysis)  I feel more tired [after dialysis]. When I started to do dialysis the same like, I’m fighting against time... The time I leave here and the time I get home because your body to see that is, it just don't stop. Once I eat and get a meal in me, I’m finna take a nap and, ... By the time I wake up it's three, four o'clock in the evening. That day belongs to dialysis, like I said. (P37, F, In-center dialysis) |
| **Entry access sites** |
| And then they couldn't get in my vein like damn. And I was admitted and they put a catheter in again and that was in from [four month timeframe], so that was disconcerting to say the least. (P7, F, In-center dialysis)  Every time I hook up, I get sick. It doesn't drain right. And every time it doesn't drain then it fills my stomach up to full. I get sick. (P17, F, Home dialysis)  I was having complications with the catheter. It wasn't just working. Oh, it was driving me crazy. So then I made a decision to have a surgery for the peritoneal catheter…And then I started my dialysis at home. (P40, F, Home dialysis) |
| **Dialyzing schedule** |
| This is so much better because it's nocturnal, and I do it overnight when I'm in bed. Then I get up in the morning, and I'm free all day. And you're not the other way with hemo. (P6, F, Home dialysis)  Selling point of doing it, it was on my time and it was that I would be able to spend time at home with my kids still and not gone most of that one day. So that was, "Hey, I feel better." (P14, M, Home dialysis) |
